# Treatment of Fracture of the Jaw, with Critical Remarks

**Published:** 1880-10

**Authors:** Thomas Brian Gunning

**Affiliations:** New York


ARTICLE III. PART V.

### TREATMENT OF FRACTURE OF THE JAW, WITH CRITICAL REMARKS.

BY THOMAS BRIAN GUNNING, D. D. S., NEW YORK.

It has been shown that, as late as the year 1875, erroneous views were sent out by those undertaking to instruct specially upon the face and jaws; and the following quotation from a book published as "the embodiment of the latest knowledge upon the different subjects treated," prove that this is done even in the present year.

In a treatise on Oral Deformities* on page 366, under "Displacement" it is written, "Garretson says: If the freed portion be the anterior or chin part, it will be dragged downward and backward by the action of the genio-hyoid, hyo-glossus, and digastric muscles.

*A Treatise on Oral Deformities as a branch of Mechanical Surgery, by Norman W. Kingsley, M. D. S., D. D. S.: D. Appleton & Co., New York, 1880.

If the neck of the bone is broken, the body is dragged forward by the action of the pterygoid, crepitation and mobility will be very apparent, and much pain will attend the movements of the jaw, produced by the displacing action of the temporalis."

This is quoted by Dr. Kingsley from a work by Professor James E. Garretson† so long known as Oral Surgeon and Clinical Lecturer to the University of Pennsylvania and now as Dean of the Philadelphia Dental College, in which he is Professor of Anatomy and Surgery, and Surgeon to the Oral Clinic. These erroneous views may therefore, mislead so many that it is very necessary to correct them. Now, however free the anterior part of the jaw or chin may be through fracture, the hyo-glossus muscle cannot displace it, for this muscle neither touches the jaw nor affects the muscles attached to this bone.

Again, if the neck of the condyle be broken, the pterygoid muscle cannot drag the body of the jaw forward any more than when unbroken; but on the contrary it is dragged *backward* by the muscles attached to the inside of the chin; assisted by the omo-hyoid, all of which act in eating and in speaking, and those especially which control the head pull the jaw strongly back.

We then read, " Bertrandi states that, when the fracture is near the angle, the smaller fragment is drawn backward by the pterygoid and masseter, the sterno-hyoid and digastric not having power enough over the lower fragment."

Bertrandi wrote, say a hundred years since, that this statement is presented at all is indefensible, it would be difficult to give one more inconsequent. In fact, the uselessness of these quotations is only equalled by their tendency to mislead the student in respect to the physiological and pathological anatomy of the parts referred to. The remainder of this section does not redeem it. I have remarked upon these extracts as "Displacement" has much in common with Diagnosis," under which heading Dr. Kingsley says,* in the two-fifths of a page which he gives to this important subject, several other things that require correction.

Now, fracture is not, as he says, generally accompanied with "a good deal of salivation." This flow of saliva may be associated with other injuries and it is not at all diagnostic of fracture.

*Op. cit. p. 368.

†A treatise on the Diseases and Surgery of the Mouth, jaws, and Associate Parts, By James E. Garretson, M. D., D. D. S.: J. B. Lippincott & Co., Philadelphia, 1869.

It is incorrect to say, "there is also displacement of fragments which the irregularity in the line of the teeth shows readily, together with contusion and laceration of integuments." Fracture may exist without any one of these symptoms; and all these, except displacement of fragments, may be present without fracture.

I now quote the whole section before remarking further.

"DIAGNOSIS.—The symptoms attending a fracture are rarely obscure. There is always unnatural mobility, generally crepitation, more or less pain, particularly at the seat of fracture, a good deal of salivation, with but little hæmorrhage. There is also displacement of fragments, which the irregularity in the line of the teeth shows readily, together with contusion and laceration of integuments.

"Where the diagnosis is difficult, particularly if fracture of the coronoid or condyloid process be suspected, the surgeon by passing the index finger into the mouth well back can, in conjunction with the other hand, so manipulate the parts as to detect the fracture should any exist. This examination should be further extended to determine if there be any dislocation, particularly if from the nature of the blow such a result would be likely to occur.

"In such cases there is almost always dislocation, or at least displacement. Should this occur, Ribes has described an excellent mode of reduction; in fact, it is the only way it can be successfully accomplished. The index finger of the right hand is carried into the mouth, and the displaced fragment searched for. With the aid of the left hand, applied externally, the piece is to be replaced and held in its normal position by forcing the jaw upward against the superior maxilla."

I have already shown that within the mouth no exploration can be made back of the anterior border of the ramus; therefore, to "so manipulate the parts to detect the fracture, should any exist," or "to determine if there be any dislocation;" the body of the jaw whether it contains teeth or not is, in addition to the ramus, the only part which can be manipulated, and to do even this, a finger or thumb must generally be under the jaw outside the mouth to oppose the finger or thumb which rests within upon the teeth, etc.

In this way the front of the jaw is so held that the ascending rami can be moved readily in any direction, and if fracture exists, crepitation between the ends of the fragments will indicate it with more certainty than muscular movements, in which the friction of the soft parts frequently misleads.

It has taken some space to point out the errors in Dr. Kingsley's twelve lines on diagnosis, between which, and the close of the section which in fact, is devoted to the *reduction* of fracture, there are suggestions as to dislocation of the condyle which he intimates is likely to result from blows of some sort. These require attention especially as in the last two lines on page 363, when speaking of the special sources of protection from fracture of the ramus, he says, refering to one, "and by the case with which the fracture might slip, and thus break the force of the blow."

But the ease with which the articulation *might* slip, affords no protection, and if fracture of the ramus ever has been averted by the laxation of the condyle, which is very doubtful. I do not know a case which justifies such an opinion.

A blow upon the front of the jaw could hardly fracture the ramus, as the neck of the condyle would break from the sigmoid notch, rather than the wider part of the ramus below it. The ramus is therefore protected from fracture through blows from the front by the neck of the condyle, not by dislocation. The effect of a blow on the side of the ramus cannot be averted by the dislocation, for the condyle and cartilage, are prevented from going in by the depth of the glenoid cavity in this part, to which is superadded the descending spine of the sphenoid bone and a strong supporting ridge which springs from the temporal bone, and which also protects the internal caroted artery, as it enters the skull. Therefore, while dislocation cannot be caused by blows on the flat side of the ramus, its fracture is very probable, for as before shown the bone is likely to give way near the inferior dental foramen.

Even if the mouth were open when the blow fell upon the ramus, although fracture might follow, dislocation could not; for the condyle would still be firmly supported against the lateral blow; while if the chin were struck, the temporal and other muscles would draw the condyle up and back into place. Luxation of the condyle which occurs in yawning and vomiting, is caused by contraction of the external pterygoid and digastric muscles in function; when it follows upon opening the jaw wide, for operations in the mouth, etc., the relaxation of the muscular action is voluntary, or if under ænesthetics, it is suspended. But when the jaw is exposed to fracture through blows or other violence, the muscles are on the alert and rarely fail to draw the condyle into place.

These explanations show that the articulation of the condyle with the glenoid cavity is not so weak or unprotected that it gives way and saves the ramus from fracture; it is, however, proper to say, that it is rather in connection with fracture that Dr. Kingsley says: "There is almost always dislocation or at least displacement." He, however, does not clearly show in what class of cases "there is almost always dislocation or at least displacement."

The fact is that dislocation of the condyle of the jaw is very rarely associated with fracture in any part of the bone; I have no recollection of seeing it. In case 6, the right condyle was dislocated outward and backward, but it was put in place before I was called in. Now dislocation occurs in connection with fracture so seldom, that Mr. Christopher Heath, F. R. C. S., in his admirable work, "*Injuries and Diseases of the Jaws*," could present only four, including this one which he quoted from my paper on "Interdental Splints," (New York Medical Journal, 1866). Those displacements of the condyle which are associated with fracture of its neck or of the socket, cannot, in my judgment, be properly classed as dislocations.

Dr. Kingsley's sweeping endorsement of Ribes' proceedure in the case of the Cannonier, whose cheek being shot away, no obstacle prevented the finger from reaching the pharynx without movement of the jaw, recalls the fact that he is President of the Board of Censors of the State of New York, and those asking permission to practice are examined by him on the treatment of fracture of the jaw. Notwithstanding that this special duty calls for full knowledge of the parts involved, and affords many opportunities for acquiring experience, he gives directions in his book in respect to the condyle which are impracticable for reasons given in correcting Malgaigne and Hamilton, and more especially through the *action* of the *external pterygoid* muscle which *pulls* the condyle forward and holds it displaced, while the mouth is open in order that the finger may get into the pharynx. Consequently, even if the condyle could be examined from the inside it could not be controlled, for the muscular action would displace it the moment the finger was removed to force the ramus up against the fractured surface.

In the case of C. B., fracture in the socket of the right canine tooth allowed the jaw to fall back so much as to lead the surgeons to su p pose the necks of the condyles had given way; and in the case which follows, the displacement of the jaw bodily, through strain of the ligaments and injury to teeth was followed by treatment for fracture in the necks of the condyles; although the jaw was not broken in and place. Again, in case 4, a cut in the left masseter muscle caused displacement, which led the surgeons in the Bellevue Hospital to suppose the neck of the condyle was broken. To these three causes of error in Diagnosis, I give one more abnormal condition, which might easily mislead.

A patient under my treatment for 40 years, whose teeth in both jaws were unusually regular, and the lower teeth articulated to the upper ones perfectly for the whole of 39 years, was then attacked by rheumatism so severely that it was supposed she would remain helpless, like her mother through the last years of her life. Ten months after, however, I was agreeably surprised by seeing her recovering so far as to get up into my chair without assistance. On looking at her teeth I found the lower front incisors could not be brought forward and up against the upper incisors. They were kept apart nearly three-eighths of an inch by the back teeth meeting to soon, the condyle going up too far into the glenoid cavities, through alterations in their articulations. The patient was 57 years old and very deaf. The parts in front of the ears however, had never given any trouble, but there was marked shrinkage of the integuments and loss of plumpness generally. This change in the jaw came in a few months, and if associated with a fall or blow might be misunderstood. The jaw had never been dislocated nor its movements interrupted in the least. A fracture in the symphysis will allow of much spreading of the angles, but on looking into the mouth the injury might be undiscovered. In one case I saw the symphysis fractured so exactly that the adjoining teeth remained firm; while the gum was healthy. The patient 24 years old had lost one central incisor to give room, but the other stood out in front of the lateral precisely in the symphysis. When the man fell on his chin, this small wedge shaped tooth struck against the upper teeth with such force that it was driven into its socket; this split the halves of the lower jaw apart, and the tooth was removed by the fingers.

Fracture in any part of the jaw between the canine teeth, and in or near the socket of either will allow the jaw to be carried back by the muscles and especially if the patient is robust. If no injury can be seen within the mouth and the angles are thrown out so far, that on closing the teeth up, those in the front will not reach out to their usual place against the upper teeth, firm pressure upon each angle of the jaw, may reveal the seat of fracture by causing pain even when crepitation is absent and if the injury is so recent that adhesions have not formed the angles by going in will force the front of the jaw forward and bring the teeth into place.

If the injury is not quite recent the condition of the patient from concussion of the brain, or other injury; even debility alone may have been such that the displaced bone is firmly united, and the case presents a serious complication of the first injury. This displacement may be the result of sleeping on the side; whether the jaw was bandaged or not; any one of these conditions may counteract the muscular displacement, or on the other hand increase it. The suggestions in regard to the effect upon recent fracture of the displacement of the injury being maintained through impractition of the fragments, and also of any injury to the muscles or ligatures, are of course pertinent when examining those of older injuries, and should be borne in mind. These varied conditions however, only require intelligent and careful examination to prevent them from misleading the surgeon.

These remarks upon diagnosis of fracture of the jaw are not given as complete or exhaustive, they have been called forth rather by the apparent need of information upon special aspects of the subject which is clearly shown in the works of the several writers, who, within the last twenty years have been foremost as instructors in the surgical and dental schools of the United States. In their writings much is given that may assist the student, while in my own views of treatment of fracture of the jaw to which I now pass, those which I have submitted will be fully sustained by the results in well known cases.

### TREATMENT OF FRACTURE OF THE JAW.

The surgeon called to any injury of the head or face should, if the jaw is hurt, see whether it will move properly and close the lower teeth against the upper ones as before the accident; and this even when it is at once seen that the teeth are displaced. When the muscular action does not adjust the parts, he should assist or do it with his fingers; for if this is tried soon after fracture of the body of the jaw the parts should go into place temporarily, at least. If, however, this does not reduce the fracture it is impacted, or the first displacement is in some other way maintained, and the fragments should be taken firmly by the thumb and fingers of each hand and so forced apart as to allow the broken surfaces to be set right. A dry towel held between the hands and the wet tissues will give firmer hold. Muscular action should be avoided by the patient.

The relief afforded when the sharp fragments are in place is always marked and often singularly great.

The surgeon should therefore release the parts at once from the displacement of the accident, unless the patient's condition is so bad that fatal consequences might ensue from any increase of suffering before partial recovery from the shock.

Insensibility from concussion affords a most favorable opportunity not only to set the fracture but to apply the splint.

It is, however, sometimes very difficult to replace the fractured parts, especially if they have been left unset for several days.

In August, 1873, a girl about ten years old when running in the Bath road, Newport, came in contact with a horse, and her jaw broke between the left incisor teeth. Several physicians were called and two saw the case on different days, but accomplished nothing. One formerly attached to the New York Hospital spoke of it to Dr. Austin L. Sands, and he advised that I should be called In. I saw the patient late in the second day after the accident, and to my surprise found the left fragment within and above the right, and firmly held the soft parts, having partially united.

The child was suffering terribly, whereas, had this displacement been reduced immediately after the accident, she would have been comparatively easy, even if nothing had been done to keep the bone steady. It required all the strength of my fingers to wrench the parts asunder. During this minute or two of extreme suffering the child made no resistance, feeling assured that now she was going to be relieved. A pad was placed between the teeth of the left side, to keep the elevated fragment down, and a four-tailed bandage worn around the chin and head until the next morning. The patient was then much relieved, and impressions of both upper and lower teeth were taken, as the growth between the fractured surfaces made it advisable to set the fracture by means of the splint. The upper plaster cast was therefore required to set the lower cast right, in order that it might mould the rubber splint.

I had no apparatus in Newport to make the splint, but Dr. C. A. Bracket kindly allowed it to be made in his laboratory.

The splint was a simple shell of hard rubber which fitted all the teeth of the lower jaw, and was tied to an infant molar on each side. The top of the splint took the place of the lower teeth in eating, the jaw moving naturally.

Dr. Brackett was much interested in the treatment, having seen nothing like it before. He had graduated from the Dental School of Harvard University where Garretson's Surgery of the mouth was used, but this book, although showing my cuts, leads the reader to suppose my splint is used with a bandage around the head ; whereas, my splints are intended to obviate the use of these vicious appliances.

I returned to New York on September 1st, but left the case in Dr. Brackett's care.

The splint was applied August 18th ; by September 8th the fracture had united well, but as the two incisor teeth were still weak the splint was worn without ligatures until October 1st.

The unaided fingers are sometimes unable to bring the fragments into place ; in case 6 I found it necessary to pass pack-thread around the teeth, with a piece of wood to draw by, yet the fracture was but four days old.

*To be continued.*

